# Local and Metastatic Relapses in a Young Woman with Papillary Squamous Cell Carcinoma of the Uterine Cervix

**DOI:** 10.3390/diagnostics12030599

**Published:** 2022-02-26

**Authors:** Ha Young Woo, Hyun-Soo Kim

**Affiliations:** 1 Department of Pathology, National Cancer Center, Goyang 10408, Korea; beliefi31@gmail.com; 2 Department of Pathology, Kyung Hee University Hospital, Kyung Hee University College of Medicine, Seoul 02447, Korea; 3 Department of Pathology and Translational Genomics, Samsung Medical Center, Sungkyunkwan University School of Medicine, Seoul 06351, Korea

**Keywords:** cervix, papillary squamous cell carcinoma, recurrence, metastasis

## Abstract

Papillary squamous cell carcinoma (PSCC) is a rare histological type of cervical carcinoma whose biological behavior has not been fully established. A 33-year-old woman with an exophytic cervical mass underwent radical hysterectomy and bilateral pelvic lymph node dissection. Histological examination of the tumor revealed numerous papillary fronds lined by atypical stratified squamous cells, resembling high-grade squamous intraepithelial lesions or urothelium. She was diagnosed with stage IB1 PSCC. Three months postoperatively, a 5.7 cm vaginal stump mass was detected. She received chemoradiotherapy, which helped her achieve a complete response. However, nine months postoperatively, she developed pelvic lymph node metastases. We present a rare case of recurrent cervical PSCC in a young woman. PSCC of the uterine cervix can recur rapidly within just a few months and become aggressive, as in the present case.

Cervical carcinoma is one of the most common types of carcinoma that cause mortality in women worldwide [1,2,3]. The most common histological type is squamous cell carcinoma (SCC) (75%), followed by adenocarcinoma (10–25%), adenosquamous carcinoma, and neuroendocrine carcinoma [1]. Papillary SCC (PSCC) is a rare variant of cervical SCC [4,5,6], accounting for 1.6% of all cervical carcinoma cases [7]. PSCC is histologically characterized by an exophytic architecture consisting of thin or broad papillary fronds lined by stratified squamous epithelium that resemble high-grade squamous intraepithelial lesions (HSILs) or urothelium [8,9]. The biological behavior of cervical PSCC has not been fully established because of the rarity of the disease. This report presents a rare case of cervical PSCC occurring in a young woman who developed local and metastatic relapses.

A 33-year-old woman presented with an exophytic cervical mass. She had a history of abnormal cervicovaginal cytology and high-risk (type 16) human papillomavirus infection. Punch biopsy results were consistent with SCC. Preoperative magnetic resonance imaging (MRI) revealed an exophytic mass located in the right lateral aspect of the uterine cervix (Figure 1; upper panel). The mass seemed to protrude into the vaginal lumen. Positron emission tomography (PET) revealed a focal hypermetabolic lesion in the cervix. No abnormal uptake was observed elsewhere in the body. She underwent radical hysterectomy with bilateral pelvic lymph node dissection. Figure 2 illustrates the gross appearance of the tumor. Histological examination revealed primary cervical SCC with 16 mm as the greatest dimension and 1 mm as the deepest invasion depth (Figure 2). The histological differential diagnoses included HSIL, verrucous carcinoma, and transitional cell carcinoma [7,8,10,11]. However, an overt stromal invasion, moderate-to-severe nuclear pleomorphism, and cytokeratin 20 negativity on immunostaining excluded those entities (Figure 3). The initial International Federation of Gynecology and Obstetrics (FIGO) tumor stage was IB1.

Three months after surgery, MRI and PET revealed a 5.7 cm recurrent mass in the vaginal stump (Figure 1; middle panel). Punch biopsy showed morphological findings similar to the primary cervical SCC. After the patient had undergone chemoradiation therapy for three months, imaging studies revealed complete remission of the vaginal tumor. However, the follow-up MRI nine months postoperatively showed enlarged right pelvic lymph nodes. PET also detected hypermetabolic lymph nodes in the right obturator area (Figure 1; lower panel).

We described a rare case of recurrent cervical PSCC occurring in a 33-year-old woman. She was initially diagnosed with early-stage disease (FIGO stage IB1). Since there was no lymphovascular space invasion and all resection margins were tumor-free, she did not receive postoperative adjuvant treatment. Three months postoperatively, the tumor relapsed locally at the vaginal stump. She underwent chemoradiation therapy and achieved complete remission. However, pelvic lymph node metastases were detected nine months postoperatively.

The frequency of cervical PSCC is known to be approximately 1.6% of all cervical carcinoma cases. Considering the unpublished or unrecognized cases, the real number of cervical PSCC cases is estimated to be much higher. Our thorough literature search revealed 93 previously published PSCC cases in which the patients underwent surgical resection, and their survival data were available [4,5,6,7,12,13,14,15,16,17]. Of these, four (4.3%) patients developed recurrences, and three (3.2%) patients died of disease, with a mean follow-up period of 26 months. It remains unclear whether PSCC of the uterine cervix represents a different group of tumors or is morphologically similar within a single homogeneous entity. According to previous reports, cervical PSCC has similar or less aggressive behavior than conventional SCC [6,7,8]. This suggests that PSCC is likely a favorable prognostic group. However, the patient in the present case who had a tumor with the typical histological features of PSCC developed local and metastatic recurrences. Our observations were consistent with those of Gitas et al. [4], who reported a case of cervical PSCC that metastasized to the ovary. Al-Nafussi et al. [18] also stated that this type of tumor has a propensity for local recurrence and late metastasis. In particular, PSCC is notorious for underestimation of stromal invasion and misinterpretation as HSIL in small biopsy specimens [19]. Therefore, pathologists diagnosing cervical biopsy should make an accurate diagnosis with the possibility of PSCC in mind. PSCC of the uterine cervix can present at an advanced stage despite histological findings of a superficial invasion or early lesion, as in the present case. Although rare, local and metastatic recurrence should be monitored in patients with cervical PSCC, and an accurate diagnosis is critical.

## Figures and Tables

**Figure 1:**
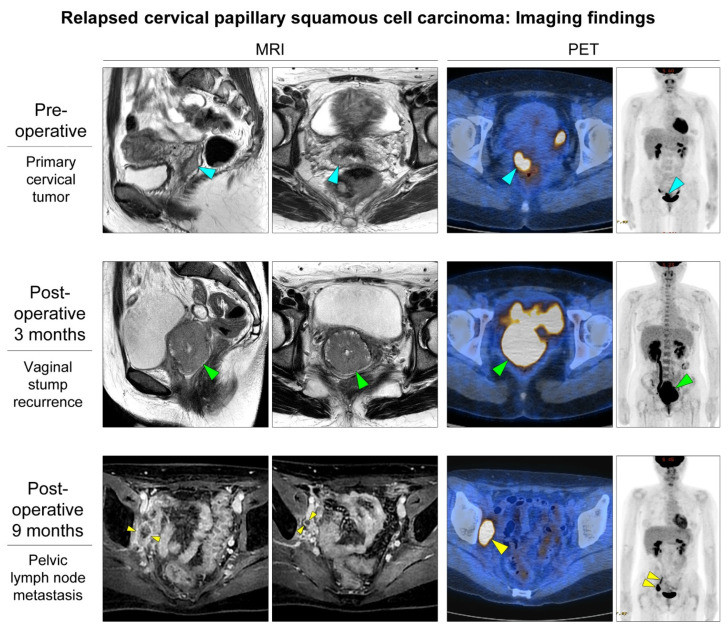
Imaging findings of a 33-year-old woman with a history of abnormal cervicovaginal cytology and high-risk human papillomavirus infection. Preoperative sagittal and axial magnetic resonance imaging (MRI) reveal an exophytic cervical mass (blue arrowheads) that seem to protrude into the vaginal lumen. The parametrium, lymph node, and adnexa are unremarkable. Positron emission tomography (PET) reveal a hypermetabolic lesion in the right lateral aspect of the uterine cervix (blue arrowhead), which is also shown in the maximum intensity projection image. She underwent radical hysterectomy with bilateral pelvic lymph node dissection and was diagnosed with early-stage cervical squamous cell carcinoma. Three months postoperatively, follow-up MRI and PET detect a recurrent tumor on the anterior wall of the vaginal stump (green arrowheads). She received chemoradiation therapy and achieved complete remission. However, pelvic lymph node metastases are developed nine months postoperatively (yellow arrowheads). Axial MRI and PET show two enlarged and hypermetabolic lymph nodes in the right obturator area.

**Figure 2:**
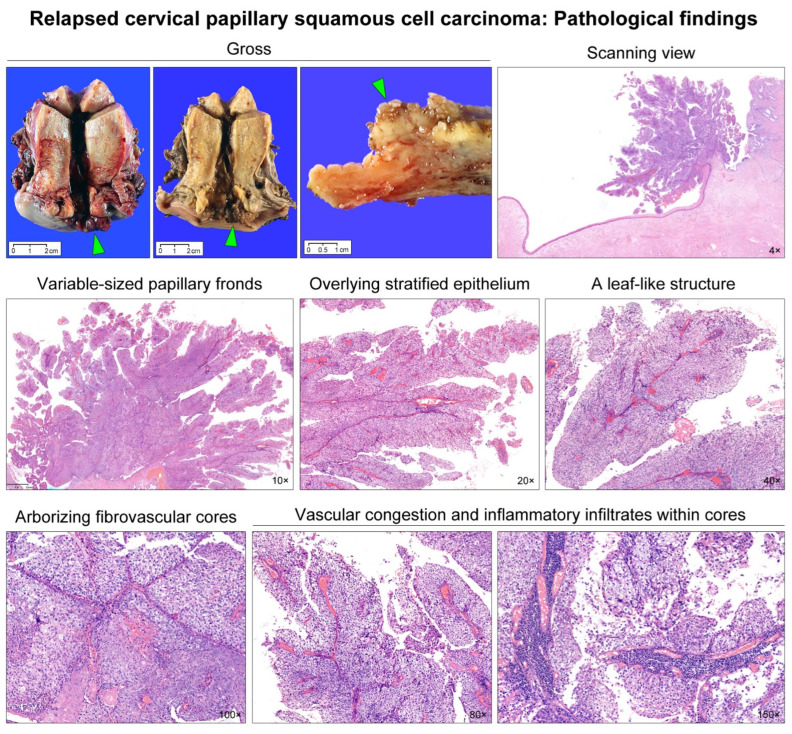
Gross and microscopic findings. Gross examination reveal an exophytic tumor (green arrowheads) arising from the right lateral aspect of the uterine cervix and protruding into the vaginal lumen. The cut surface show a papillary-appearing tumor (green arrowhead) with unapparent stromal invasion. On scanning magnification, the tumor is characterized by a predominantly exophytic growth towards the vaginal lumen. Numerous, variably-sized papillary fronds are richly vascularized and lined by stratified squamous epithelium. Finger-like protrusions or leaf-like structures and arborescent fibrovascular cores are frequently observed. The tumor papillae are either thin with a delicate fibrovascular core or broad with occasional vascular congestion and lymphoplasmacytic infiltrates. These histological features are consistent with papillary squamous cell carcinoma of the uterine cervix.

**Figure 3:**
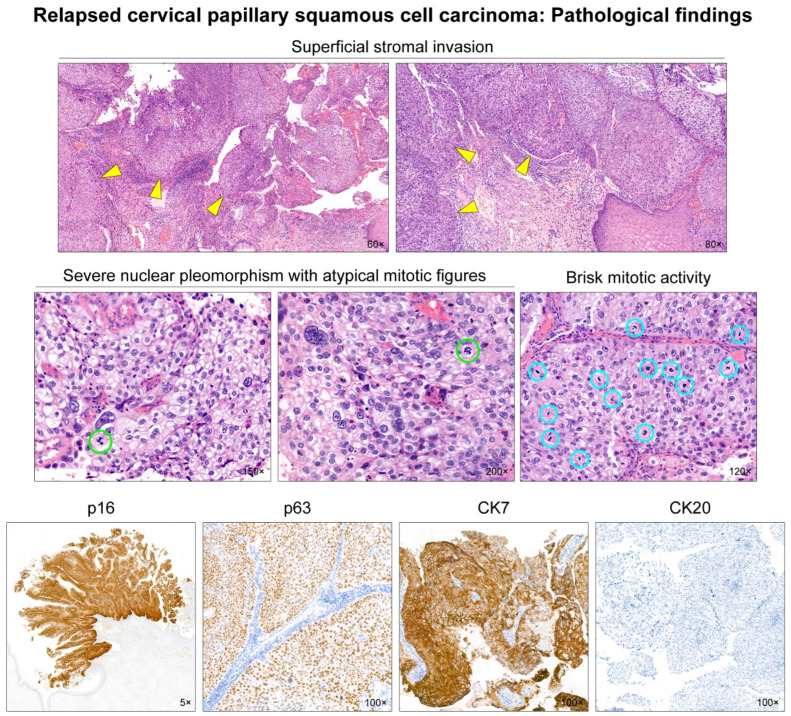
Microscopic findings and immunostaining results. A few microscopic foci (yellow arrowheads) of superficial stromal invasion are noted. The invasion depth measures 1 mm at the deepest point. The tumor cells demonstrate moderate-to-severe nuclear pleomorphism accompanied by occasional atypical mitotic figures (green circles). Mitotic activity is brisk (blue circles). Immunohistochemically, the tumor exhibits block p16 positivity and diffuse and strong p63 expression, supporting the diagnosis of human papillomavirus-associated squamous cell carcinoma. Cytokeratin 7 (CK7) is uniformly and intensely positive for the tumor cells, whereas CK20 is completely negative, indicating this tumor is a variant of squamous cell carcinoma.

## Data Availability

Not applicable.
